# Macrophages, dendritic cells, and regression of atherosclerosis

**DOI:** 10.3389/fphys.2012.00286

**Published:** 2012-07-18

**Authors:** Jonathan E. Feig, Jessica L. Feig

**Affiliations:** ^1^Zena and Michael A. Wiener Cardiovascular Institute, Mount Sinai Medical CenterNY, USA; ^2^Department of Medicine, New York University Medical CenterNY, USA

**Keywords:** atherosclerosis regression, CCR7, dendritic cells, HDL, LXR, macrophages

## Abstract

Atherosclerosis is the number one cause of death in the Western world. It results from the interaction between modified lipoproteins and cells such as macrophages, dendritic cells (DCs), T cells, and other cellular elements present in the arterial wall. This inflammatory process can ultimately lead to the development of complex lesions, or plaques, that protrude into the arterial lumen. Ultimately, plaque rupture and thrombosis can occur leading to the clinical complications of myocardial infarction or stroke. Although each of the cell types plays roles in the pathogenesis of atherosclerosis, the focus of this review will be primarily on the macrophages and DCs. The role of these two cell types in atherosclerosis is discussed, with a particular emphasis on their involvement in atherosclerosis regression.

## Macrophages and atherosclerosis

Atherosclerosis, a chronic inflammatory disease that occurs within the artery wall, is one of the underlying causes of vascular complications such as myocardial infarction, stroke, and peripheral vascular disease. Atherogenesis is a process that occurs over many years with the initiation phase being the subendothelial accumulation of apolipoprotein B-containing lipoproteins (ApoB) (Williams and Tabas, 1995; Glass and Witztum, 2001; Williams et al., 2007, 2008). These particles undergo modifications, including oxidation and hydrolysis, leading to the activation of endothelial cells. These cells secrete chemoattactants called chemokines that interact with specific receptors expressed on monocytes essentially “recruiting” the cells into the lesion. The monocytes then roll along the endothelial cells via interactions of specific selectins, [i.e., P-selectin glycoprotein ligand-1 (PSGL-1)] with attachment being mediated by monocyte integrins such as very late antigen-4 (VLA-4) and lymphocyte function-associated antigen 1 (LFA-1) to the respective endothelial ligands vascular cell adhesion molecule-1 (VCAM-1) and intercellular adhesion molecule-1 (ICAM-1) (Glass and Witztum, 2001; Mestas and Ley, 2008). Once attached, a process called diapedesis occurs by which monocytes enter the subendothelial space (Kamei and Carman, 2010). Having accessed the subendothelial space, recruited monocytes differentiate into macrophages, a process driven by interactions with the extracellular matrix (ECM) and cytokines, including macrophage colony-stimulating factor and members of the tumor necrosis factor family (Johnson and Newby, 2009). The uptake of oxidized LDL by the macrophages occurs via scavenger receptors, notably the type A scavenger receptor (SRA) and CD36, a member of the type B family (Kunjathoor et al., 2002). Interestingly, deletion of SRA or CD36 doesn't ameliorate plaque development suggesting that other mechanisms must be present (Moore et al., 2005; Goyal et al., 2012). Indeed, fluid phased pinocytosis was shown to play a role in foam cell formation (Kruth et al., 2005)

The cholesteryl esters of the apoB particles that are ingested are hydrolyzed into free cholesterol, which occurs in late endosomes. The free cholesterol is then delivered to the endoplasmic reticulum (ER) where it is re-esterified by acyl-CoA: cholesterol ester transferase (ACAT) (Maxfield and Tabas, 2005). It is this process that leads to the macrophages having the “foamy” appearance. Data from *in vivo* studies indicate that the form of ACAT in macrophages, ACAT1, contributes to foam cell formation in the arterial wall and the development of atherosclerosis. Studies in apoE^−/−^ mice, however, have suggested that complete deficiency of ACAT1 activity is not anti-atherogenic, in part because of toxicity resulting from adverse effects on tissue cholesterol homeostasis (Rudel et al., 2001). Hence it was tested whether partial inhibition of ACAT1 and ACAT2 (expressed in liver and intestine) activities reduces atherosclerosis development in apoE-deficient mice and avoids toxicity. Indeed, partial inhibition led to a reduction in atherosclerosis (Kusunoki et al., 2001). In order to extend these studies in humans, a prospective, randomized, double-blind, placebo-controlled study (Carotid Atherosclerosis Progression Trial Investigating Vascular ACAT Inhibition Treatment Effects [CAPTIVATE]) was designed. It included 892 patients heterozygous for familial hypercholesterolemia. Interestingly, pactimibe (ACAT inhibitor) had no effect on atherosclerosis as assessed by changes in maximum carotid intima-media thickness (CIMT) compared with placebo but was actually associated with an increase in mean CIMT as well as increased incidence of major cardiovascular events (Meuwese et al., 2009). Whether these results were truly due to this specific compound or is a class effect is not entirely known. Future investigative work will need to be performed in order to determine whether there is a future in ACAT inhibitors as therapies against plaque formation.

It is well-known that macrophages contribute to formation of the necrotic core and fibrous cap thinning that characterizes the vulnerable plaque (Virmani et al., 2002a,b; Tabas, 2010a). How do these macrophages ultimately contribute to the vulnerable plaque? Macrophage-derived matrix metalloproteinases (MMPs) are a family of proteins that can degrade various types of ECM and hence promote rupture. Moreover, once activated, certain MMPs can activate other ones (Tabas, 2010a). Studies have shown a temporal and spatial correlation between the presence of macrophages in rupture-prone shoulder regions of plaques, thinning of the fibrous cap in these regions, and local accumulation of activated MMPs, especially MMP-2 and MMP-9 (Galis et al., 1994). Since mouse models of atherosclerosis do not recapitulate plaque rupture, investigations have focused on endpoints such as lesion area or plaque collagen content. For example, increases in plaque collagen were observed when MMP-13 or MMP-14 were deleted globally or in macrophages (Deguchi et al., 2005; Schneider et al., 2008). In a study that attempted to look directly at plaque disruption, macrophage over-expression of wild-type (WT) MMP-9 did not lead to a phenotype (plaque fissuring) in apoE^−/−^ deficient mice due to a lack of MMP activation in plaques, but over-expression of a constitutively active mutant form of MMP-9 resulted in plaque fissuring (Gough et al., 2006). New mouse models of true plaque rupture will be needed in order to validate these concepts and help guide whether MMP inhibition in humans is a worthwhile strategy.

Another potential mechanism of how macrophages may promote plaque thinning and increase vulnerability is via causing smooth muscle cell (SMC) apoptosis. Vulnerable plaques show evidence of SMC death and decreased numbers of SMCs. *In vitro* data show that macrophages can trigger apoptosis in SMCs by activating their Fas apoptotic pathway as well as by secreting pro-apoptotic TNFα and nitric oxide (Boyle et al., 2003). Studies demonstrated that in regions of vulnerable plaques that have defective clearance of apoptotic cells, macrophage secretion of TGFβ may be decreased hence depriving neighboring SMCs of this important inducer of collagen synthesis (Fadok et al., 1998). Even after plaque rupture, the macrophage continues to play a role as it secretes prothrombotic tissue factor thereby accelerating thrombus formation (Glass and Witztum, 2001; Williams et al., 2007, 2008).

Another critical feature of vulnerable plaques is the necrotic core. Necrotic cores arise from the combination of apoptosis of advanced lesional macrophages and defective phagocytic clearance (efferocytosis) of the apoptotic macrophages in advanced plaques (Tabas, 2010a). Studies have shown that necrotic cores contribute to inflammation, thrombosis, proteolytic plaque breakdown, and physical stress on the fibrous cap (Virmani et al., 2002a,b; Tabas, 2010a). Growth factor deprivation, oxidative stress, and death receptor activation by ligands are likely processes that contribute to macrophage death in advanced atheromata. Recent studies, *in vivo*, have recently shown that prolonged activation of ER stress pathways, primarily the unfolded protein response (UPR), contributes to macrophage death and subsequent plaque necrosis in advanced atheromata (Tabas, 2010b, 2011; Tabas and Ron, 2011). It is important to note that macrophage apoptosis by itself will not trigger plaque necrosis. Rather, plaque necrosis results when apoptotic macrophages are not sufficiently cleared by phagocytes (Tabas, 2010a). Efferocytosis of apoptotic macrophages is important because it leads to protective effects such as clearing the cells before membrane damage leads to leakage of toxic intracellular material. Several possible mechanisms might contribute to defective efferocytosis, such as oxidative stress-induced efferocyte death resulting from defective cholesterol efflux after apoptotic cell engulfment and protease-mediated cleavage of the efferocytosis receptor MerTK (Sather et al., 2007; Yvan-Charvet et al., 2010). Additional studies will need to be performed in order to further dissect the mechanisms involved.

It has previously been shown that there is macrophage heterogeneity in atherosclerotic plaques and both M1 as well as M2 macrophages have been shown to exist in atherosclerotic lesions (Bouhlel et al., 2007; Johnson and Newby, 2009). Polarized activated M1 macrophages are induced by interferon-gamma (IFN-γ) alone or in concert with microbial stimuli such as lipopolysaccharide (LPS). M1 cells have an interleukin IL-12^high^, IL-23^high^, IL-10^low^ phenotype and are proficient producers of effector molecules such as reactive oxygen and nitrogen intermediates. In addition, M1 cells produce inflammatory cytokines and contribute as inducer and effector cells in polarized Th1 responses. In contrast, the alternative M2 form of macrophage activation is a generic name used for various forms of non-classically activated macrophages (anti-inflammatory class) resulting from cell exposure to IL-4 or IL-13, immune complexes, IL-10, glucocorticoid, or secosteroid (vitamin D_3_) hormones. The various forms of M2 macrophages share an IL-12^low^ and IL-23^low^ phenotype as well as generally displaying high levels of scavenger, mannose, and galactose-type receptors. Importantly, arginine metabolism is shifted to the production of ornithine and polyamines via arginase. In other words, M1 macrophages are potent effector cells that produce primarily proinflammatory cytokines, such as tumor necrosis factor-alpha (TNF-α), IL-6, and IL-12. In contrast, M2 macrophages dampen these inflammatory responses by producing anti-inflammatory factors such as IL-10, transforming growth factor-beta (TGF-β), and IL-1 receptor antagonist (IL-1Ra) (Mantovani et al., 2002, 2005).

## Dendritic cells and atherosclerosis

It is now well accepted that atherosclerosis is a chronic disease of the arterial wall involving both innate and adaptive immunity (Dumitriu and Kaski, 2011). Dendritic cells (DCs) are a heterogeneous population of bone marrow-derived immune cells that specialize in capturing, processing and presenting antigens to T lymphocytes in order to induce and control immunity. DCs are morphologically characterized by the presence of several thin cytoplasmic processes (dendrites) and by large cytoplasmic veils that are continuously extended and retracted. DCs are very efficient at internalizing antigens either by phagocytosis or by receptor-mediated endocytosis. Subsequently, DCs display a fragment of the antigen, bound to a class II major histocompatibility complex (MHC), on their membrane. CD4+ T cells recognize the antigen-class II MHC molecule complex on the DC membrane. DCs then produce a co-stimulatory signal such as B7-1 or B7-2 ultimately activating the CD4+ T cells (Banchereau and Steinman, 1998; Mallat et al., 2009; Koltsova and Ley, 2011; Manthey and Zernecke, 2011; Van Vre and Bult, 2011; Van Vre et al., 2011).

It is important to note that DCs are actually present in healthy arteries and have been documented in the subendothelial space and at the media-adventitia junction (Ma-Krupa et al., 2004; Pryshchep et al., 2008). The localization of DCs adjacent to the vasa vasorum allows for the monitoring of important access pathways to the vessel wall, to present autoantigens such as oxidized LDL – CD4+ T cells, and to locally initiate inflammatory responses (Ma-Krupa et al., 2004; Pryshchep et al., 2008; Niessner and Weyand, 2010). Interestingly, DCs and T cells are arrayed in clusters that primarily locate to the shoulder and rupture prone regions of the plaque (Yilmaz et al., 2004; Bobryshev, 2005; Erbel et al., 2007). Importantly, it has been shown that patients with angina and acute MI have reduced circulating blood-derived DC precursors. In fact, blood derived DCs and plasmacytoid DCs are actually diminished in patients with angiographically documented CAD potentially due to increased recruitment to plaques (Van Vre et al., 2006; Yilmaz et al., 2006; Van Vre et al., 2010).

The identification of DCs in the arteries of animal models has facilitated the investigation of the impact of DCs in atherosclerosis (Bobryshev et al., 1999, 2001; Ozmen et al., 2002). Increased numbers of DCs have been observed in atherosclerotic lesions in mouse models of atherosclerosis (Galkina et al., 2006; Liu et al., 2008; Weber et al., 2011). Ludewig et al were the first to report a link between immune-mediated arterial inflammation and cholesterol-induced atherosclerosis mediated by DCs in a hypercholesterolemic transgenic mouse model using the defined expression of the microbial antigen β-galactosidase (β-gal) in arterial SMCs in apoE^−/−^ mice. Experimentation revealed that hypercholesterolemia selectively enhanced and perpetuated arterial inflammation. Furthermore, arterial inflammation significantly increased the susceptibility of the arterial wall to cholesterol-dependent atherosclerosis (Ludewig et al., 2000).

It was also demonstrated that dyslipidemia associated with atherosclerotic disease alters DC function: hyperlipidemia inhibits DC migration with HDL restoring it (Angeli et al., 2004). DCs can maintain antigen-processing and antigen-presenting capabilities, which allow them to efficiently prime CD4+ T cells under hypercholesterolemic conditions (Packard et al., 2008). Studies demonstrated that the peroxisome proliferator activated receptor gamma (PPAR-γ agonist ciglitazone inhibited the ox-LDL-induced maturation and immune functions of DCs (Luo et al., 2004). Interestingly, it was shown that the PPAR-α agonist fenofibrate, inhibited the ox-LDL-induced immune maturation of DCs (Shi et al., 2008). These effects of PPARs may partially explain their ability to slow atherosclerosis progression and reduce the risk of coronary heart disease independently from their metabolic effects.

Statins, HMG–CoA reductase inhibitors, are a mainstay in the treatment of hyperlipidemia. Studies have shown that these agents also possess powerful pleiotropic effects that are independent of their cholesterol lowering properties. The major effect of statins is the inhibition of cholesterol and isoprenoid synthesis, which ultimately results in upregulation of endothelial nitric oxide synthase (eNOS), an enzyme responsible for vascular endothelial function (Liao, 2002). Additionally, antioxidant effects (i.e., via the decreased production of NADPH oxidase) lead to decreased amounts of reactive oxidant species in the circulation (Endres, 2005). Inflammatory markers such as C-reactive protein (CRP) and nuclear factor κB (NF-κB) have also been shown to be reduced by statins, leading to the hypothesis that statins possess anti-inflammatory properties (Li et al., 2010). Other proposed mechanisms include immunomodulation, normalization of sympathetic outflow, plaque stabilization, decreased activation of the blood coagulation cascade, and inhibition of platelet aggregation (Mihos and Santana, 2011). Pre-incubation of lipolysaccharide-stimulated DCs with statins significantly suppressed secretion of proinflammatory cytokines as well as the ability to induce T cell proliferation and activation (Yilmaz et al., 2006). Another study provided clinical evidence that atorvastatin at 20 mg/day for four weeks significantly reduce DCs and matrix metalloproteinase expression in the aortic wall of patients undergoing abdominal aorta replacement (Kajimoto et al., 2009). These results suggest yet another pleiotropic effects of statins that may be independent of its lipid-lowering effects.

Eicosapentaenoic acid (EPA), another agent known to possess beneficial effects in cardiovascular disease, has recently been shown to promote regression of atherosclerotic lesions. The data suggests that this was in part due to the ability of the compound to modulate DCs. Flow cytometric analysis revealed that EPA increased immature DCs [CD11c(+) CD80(−) CD86(−)], increased the indoleamine 2,3-dioxygenase (IDO) in DCs, and decreased the number of CD4+ T cells. Importantly, in the presence of an IDO inhibitor (1-methyl-dl-tryptophan), the beneficial effects of EPA on regression were inhibited, suggesting that the effect of EPA was mainly mediated through IDO (Nakajima et al., 2011).

Interestingly, DCs were shown to be present in human unstable plaques (Yilmaz et al., 2004) and higher DC densities were found in carotid plaques from symptomatic patients as compared to those from asymptomatic patients (Kawahara et al., 2007). DCs were also described in human symptomatic in-stent restenosis (Skowasch et al., 2003) and in aortic aneurysms (Bobryshev et al., 1998). The above studies suggest that the presence of DCs is associated with progression of atherosclerosis. However, a study by Gautier and colleagues suggests that there is more to the role of DCs that may have been previously appreciated. They created a mouse model in which the lifespan and immunogenicity of DCs were enhanced by specific over-expression of the human antiapoptotic gene B-cell lymphoma 2 (hBcl-2) under the control of the CD11c promoter (Gautier et al., 2009). In either LDL receptor-deficient or apolipoprotein E-deficient backgrounds, DC-hBcl2 mice exhibited an expanded DC population associated with enhanced T-cell activation, a Th1 and Th17 cytokine expression profile, as well as elevated production of Th1-driven IgG2c autoantibodies directed against oxidation-specific epitopes. However, expansion of the DC population was unexpectedly associated with a decrease in plasma cholesterol levels and no acceleration of atherosclerotic plaque progression. Conversely, depletion of DCs in hyperlipidemic CD11c-diphtheria toxin receptor/apolipoprotein E-deficient transgenic mice resulted in enhanced hypercholesterolemia, thereby arguing for a close relationship between the DC population and plasma cholesterol levels (Gautier et al., 2009). These studies demonstrate that DCs are central to the atherosclerotic process by being directly implicated in both cholesterol homeostasis and immune response.

There are various types of DCs with distinct roles. For example, unlike conventional DCs, plasmacytoid DCs (PDC) are poor in antigen presentation and critical for type I interferon response. A recent study sheds light on the possible role that PDCs may exhibit. Administration of 120G8 mAb (antibody which recognizes PDCA-1 also referred to as bone marrow stromal cell antigen 2 [BST2], a marker specifically expressed on mouse PDCs (Blasius et al., 2006)) in LDLR^−/−^ mice led to PDC depletion resulting in plaque T-cell accumulation and enhanced plaque progression. The authors concluded that PDCs may play a protective role in atherosclerosis, possibly by dampening T-cell proliferation and activity in peripheral lymphoid tissue (Daissormont et al., 2011). Further work will be required in order to determine the therapeutic potential in modulating DC biology for the treatment of atherosclerosis.

## Atherosclerosis regression

The idea that human atheromata can regress at all is something that met considerable resistance over the years. The reason for this may have been that advanced atherosclerotic lesions in humans and in animal models contain calcification and fibrosis, characteristics that seem irreversible (Blankenhorn and Hodis, 1994; Williams et al., 2007, 2008). Regardless, the ability to induce atherosclerosis regression is a desirable clinical goal. The first interventional study demonstrating substantial shrinkage of atherosclerotic lesions was performed in cholesterol-fed rabbits over 50 years ago (Friedman et al., 1957). Animals received intravenous bolus injections of phosphatidylcholine (PC). After less than a week and a half of treatment, the remaining plaques were fewer and much smaller than initially with approximately 75% of the arterial cholesterol stores being removed. Using a variety of atherosclerotic animal models, other groups showed similar arterial benefits from the injection of dispersed phospholipids (Williams et al., 1984; Stein and Stein, 2001).

Armstrong and colleagues found that advanced arterial lesions in cholesterol-fed rhesus monkeys underwent shrinkage and remodeling during long-term follow-up after a switch to low-fat or linoleate-rich diets (Armstrong et al., 1970; Armstrong, 1976). The subsequent regression period (lasted 40 months) resulted in the loss of approximately two-thirds of coronary artery cholesterol, substantial reduction in necrosis, improvement in extracellular lipid levels and fibrosis, as well as lesion shrinkage. Success in atherosclerosis regression was again achieved in rabbits in 1976, following reversion to a normal-chow diet in combination with the administration of hypolipidemic agents (Wissler and Vesselinovitch, 1976). Decades later, a series of studies achieved shrinkage of atheromata in rabbits via injections of HDL or HDL-like apolipoprotein A-I (apoA-I) and PC disks (Miyazaki et al., 1995).

In spite of the clinical desirability to achieve regression, research into the factors that may be mediating this process has been hampered by the scarcity of appropriate animal models. The relative ease of progression studies, using apoE^−/−^ or LDLR^−/−^ mice, has led to an emphasis on this phase of the disease process, but this emphasis also highlights the relative dearth of models of regression. The similarities between atherosclerosis in humans and mice deficient either in apoE (Plump et al., 1992; Zhang et al., 1992; Nakashima et al., 1994; Breslow, 1996) or the LDL receptor (Ishibashi et al., 1993) suggest that molecular mechanisms underlying regression in these mouse models could be relevant to the reduction in plaque burden in the human population (Williams et al., 2007, 2008; Feig et al., 2009).

Plaque regression in mouse models of atherosclerosis has previously been demonstrated primarily by somatic adenoviral gene transfer (Yang et al., 1996; DeMatteo et al., 1997). Such approaches have been limited mainly because of the eventual loss of transgene expression, even with second-generation viral vectors, likely caused by immune responses directed against both the transgene product and adenoviral proteins. A new regression model was introduced which involves transplantation of either a thoracic aortic segment (Reis et al., 2001) or an aortic arch segment (Chereshnev et al., 2003) from apoE^−/−^ mice into WT recipient mice. Under the conditions of the WT mouse in which the dyslipidemia is corrected, regression is rapidly apparent (as judged by plaque CD68+ monocyte derived cell content). However, when the recipient is an apoE^−/−^ mouse, further progression is evident (Chereshnev et al., 2003; Llodra et al., 2004; Trogan et al., 2004, 2006). Using laser capture microdissection of CD68+ cells in the lesions, it was demonstrated that regression was characterized by the decrease in expression of inflammation-related genes such as VCAM-1, ICAM-1, monocyte chemotactic protein-1 (MCP-1), and TNF-α (Trogan et al., 2006). Interestingly the nuclear transcription factors liver X receptor alpha (LXRα), liver X receptor beta (LXRβ), and their downstream targets ATP-binding cassette transporter 1 (ABCA1) as well as ATP-binding cassette sub-family G member 1 (ABCG1) were upregulated in these same cells (Trogan et al., 2006).

Notably, the decrease in foam cell content was attributed to emigration of these cells from plaques to regional and systemic lymph nodes under regression, but not progression, conditions (Llodra et al., 2004; Trogan et al., 2006). The emigrating cells expressed markers of macrophages (such as CD68 and CD115) and DCs (such as CD11c) (Llodra et al., 2004; Trogan et al., 2006). Since migration of DCs to lymph nodes absolutely requires the chemokine receptor CCR7 (Forster et al., 1999), we hypothesized that it became induced in foam cells under regression conditions. Indeed, we found an increase in CCR7 mRNA and protein expression only in foam cells from the regression environment (Trogan et al., 2006) and showed the functional requirement of CCR7 for regression in our transplant model (Trogan et al., 2006). We then determined that LXR is required for maximal effects on plaque CD68+ cell expression of CCR7 as well as monocyte-derived cell egress during atherosclerosis regression in mice (Feig et al., 2010).

The induction of CCR7 is likely also related to the lipid milieu. This idea is in agreement with two recent reports. In the first, scientists demonstrated that loading THP-1 human monocytes with oxidized LDL suppresses the expression of CCR7 (Damas et al., 2007). In the second, it was reported that CCR7 expression was significantly downregulated in human carotid atherosclerotic plaques (Nickel et al., 2012). Using bioinformatics, we have identified potential sterol response elements (SREs) in the promoter region of CCR7 (Feig et al., 2011). We have found that statins, potent regulators of SRE-dependent transcription can induce CCR7 expression *in vivo* and promote regression via emigration of CD68+ cells in a CCR7 dependent manner (Feig et al., 2011). It was recently reported that both atorvastatin and rosuvastatin can promote regression of atherosclerosis as assessed by intravascular ultrasound (IVUS) (Nicholls et al., 2011). Our data suggests that activation of the CCR7 pathway may be one mechanism to explain how this may occur.

Although HDL has been shown to reduce atherosclerosis progression in mouse-based studies (Rubin et al., 1991; Rong and Fisher, 2000; Choudhury et al., 2004; Feig et al., 2008), its role in regression is also of great interest because of the important clinical implications. Badimon and colleagues attracted considerable attention in 1990 by showing regression of atherosclerotic lesions by infusions of the HDL plasma fraction in the cholesterol-fed rabbit (Badimon et al., 1990). Later studies demonstrated that intravenous infusion of apoAI and liver-directed adenoviral transfer of apoAI DNA led to plaque regression (Miyazaki et al., 1995; Tangirala et al., 1999). The rapidity of the regression process has been seen in that the infusion of recombinant apoAI milano (a natural mutant of human apoAI thought to be more effective in promoting reverse cholesterol transport) into apoE^−/−^ mice resulted in reductions in lipid and macrophage content of the lesions just after 48 h (Shah et al., 2001).

A limitation to examining directly the effects of HDL has been the difficulty in sustaining the elevation of HDL. Using our transplant model, we were able to tackle this issue (Rong et al., 2001; Feig et al., 2011). In our more recent study (Feig et al., 2011), plaque-bearing aortic arches from apolipoprotein E-deficient mice (low HDL-C, high non-HDL-C) were transplanted into recipient mice with different levels of HDL-C and non-HDL-C: C57BL6 mice (normal HDL-C, low non-HDL-C), apoAI^−/−^ mice (low HDL-C, low non-HDL-C), or apoE^−/−^ mice transgenic for human apoAI [hAI/apoE^−/−^] (normal HDL-C, high non-HDL-C). Remarkably, despite persistent elevation of non-HDL-C in hAI/apoE^−/−^ recipients, plaque CD68+ cell content decreased by >50% by one week after transplantation, whereas there was little change in apoAI^−/−^ recipient mice despite hypolipidemia. Interestingly, the decreased content of plaque CD68+ cells was associated with their emigration and induction of their chemokine receptor CCR7 (Feig et al., 2011). Furthermore, in CD68+ cells laser-captured from the plaques, normalization of HDL-C led to decreased expression of inflammatory factors and enrichment of markers of the M2 (tissue repair) macrophage state (Feig et al., 2011). These data emphasized the crucial role HDL may play in the regression process, which is consistent with a recent meta-analysis of clinical studies in which it was shown that atherosclerosis regression (assessed by IVUS) after LDL lowering was most likely to be achieved when HDL was also significantly increased (Nicholls et al., 2007).

To examine effects of HDL on plaque, infusion studies have been performed in humans. Basically, high risk patients were infused with either apoAI milano/phospholipid complexes (an artificial form of HDL) or phospholipid complexes (control) once a week for five weeks. Compared to the control group, by IVUS, there was a 4.2% decline in atheroma volume in the treated group, although there was no effect of a higher vs lower dose of the HDL (Nissen et al., 2003). In another infusion study, reconstituted HDL containing wild type apoAI was infused into high risk patients for six weeks. Although compared to the control group, there was no difference in atheroma volume by IVUS, there were statistically significant improvements in plaque characterization index and coronary score on quantitative coronary angiography (Tardif et al., 2007).

It is unlikely that regression of atherosclerosis occurs only through one mechanism. A recent report showed that netrin-1, a neuroimmune guidance cue, was secreted by macrophages in human and mouse atheroma, where it inactivated the migration of macrophages toward chemokines (such as chemokine [C-C motif] ligand 19, ligand for CCR7 (Forster et al., 1999)) linked to their egress from plaques. Interestingly, targeted deletion of netrin-1 in macrophages resulted in much less atherosclerosis in LDLR^−/−^ and promoted the emigration of macrophages from plaques (van Gils et al., 2012). These findings suggest that inhibition of netrin-1 may be one method of inducing regression of atherosclerosis. In contrast, Potteaux et al suggested that suppression of monocyte recruitment along with apoptosis accounted for the decrease in macrophage content during the regression seen with viral vector restoration of apoE in apoE^−/−^ mice (Potteaux et al., 2011). To begin addressing other possible mechanisms, we decided to perform microarray experiments in which we laser captured CD68+ foam cells from baseline, apoE^−/−^ recipients, and WT recipients. We were able to demonstrate that the two major subsets of monocytes CCR2^+^CX3CR1^+^Ly-6C^hi^ and CCR2^−^CX3CR1^+^Ly-6C^lo^ migrate out of lesions under regression conditions. However, their regression is impaired under hyperlipidemic conditions. Not surprisingly, we found a distinct molecular signature characterizing progression versus regression. When the data was subjected to pathway analysis using specialized bioinformatic tools, we found that genes related to migratory capacity like those that make up the contractile apparatus (actin and myosin subunits) were significantly up-regulated under regression conditions. These results support our previous reports that emphasized that regression is characterized by cells emigrating out of plaques.

Consistently, among all samples tested, the most highly up-regulated (~14X) gene under regression conditions was arginase I (ArgI). ArgI is a classical marker of the anti-inflammatory M2 macrophage (Mantovani et al., 2002, 2005). Although we are first to report that argI is differentially expressed under regression of atherosclerosis (Feig et al., 2011), the idea is supported by some studies in the literature. In macrophages, L-arginine can be metabolized by NOS and arginase to form NO and urea, respectively (Getz and Reardon, 2006). ArgI is induced in a number of vascular cells, including endothelial cells, SMCs, and macrophages by Th2 cytokines including IL-4, IL-10, IL-13, and granulocyte macrophage colony-stimulating factor (Munder et al., 1999; Chang et al., 2000; Jost et al., 2003). Teupser et al reported that peritoneal macrophages from an atherosclerosis-prone strain of rabbits had lower levels of argI expression (Teupser et al., 2006). To test the effect of argI over-expression on atherosclerosis susceptibility, bone marrow from argI-transgenic (expressing human argI under the control of a CMV promoter) and non-transgenic animals were transplanted into LDL-receptor deficient recipients. After 12 weeks of high fat diet, animals were sacrificed. Importantly, there were no significant differences in lipoprotein levels. However, atherosclerosis of animals transplanted with transgenic bone marrow was significantly reduced compared to controls. Hence, it was concluded that argI might constitute an interesting novel target for the prevention of atherosclerosis (Teupser et al., 2007). In addition, the group led by Newby demonstrated that foam cell formation further lowers argI levels (Thomas et al., 2007). Whether argI is protective against atherosclerosis simply by reducing nitric oxide (NO) production is an unanswered question. However, a wealth of evidence suggests that endothelial NO production (from endothelial nitric oxide synthase [eNOS]) is protective against atherosclerosis by, for example, causing vasodilatation, reducing LDL oxidation, and reducing monocyte recruitment to lesions (Landmesser et al., 2004). Macrophage NO production (most actively from inducible nitric oxide synthase [iNOS]) is most likely harmful (Luoma et al., 1998; Boyle et al., 2002), because iNOS knockout attenuates atherosclerosis in apoE^−/−^ mice (Detmers et al., 2000). It should be noted that there are species differences in terms of macrophage NO production and regulation. For example, murine macrophages produce large amounts of NO and L-citrulline from L-arginine via induction of iNOS. Human macrophages exhibit iNOS activity, albeit induced by other stimuli rather than those inducing nitrite production in murine macrophages (Fang, 2004; Schneemann and Schoeden, 2007). It is clear, though, that high levels of NO and superoxide within the lesions creates conditions favorable for formation of peroxynitrite, which may cause injury as a consequence of protein nitration (Radi, 2004). Furthermore, high levels of NO from macrophages also cause apoptosis of SMCs (Boyle et al., 2002), which could promote plaque instability. It is reasonable, then, to conclude that down-regulation of argI and the consequent up-regulation of NO production enhance the pathological potential of the foam cells. In short, our results and these other studies all point to argI being a candidate novel target for the treatment of atherosclerosis. In agreement with this hypothesis, polymorphism in the argI gene has been consistently associated with increased frequency of myocardial infarction in humans (Dumont et al., 2007). Recent reports suggested that members of the PPAR family can regulate argI expression (Gallardo-Soler et al., 2008). Given the fact that PPARs can induce LXR expression and that LXRα expression was significantly increased in the WT recipients (Trogan et al., 2006), we wondered whether there would be a relationship between LXR and the M2 phenotype. We recently reported a molecular mechanism linking LXRα to the regulation of argI via purine box factor 1 (PU.1) and interferon regulatory factor 8 (IRF8) transcription factors (Pourcet et al., 2011).

Recently, another model, the Reversa mouse, has given us the opportunity to extend our studies and determine whether the findings in the transplant model were generally applicable to the regression process (Lieu et al., 2003; Feig et al., 2011). Reversa mice (LDLR^−/−^ApoB100/100Mttpfl/flMx1Cre^+/+^) have severe hypercholesterolemia and atherosclerosis as a result of homozygosity for the LDL receptor knockout allele and an “apoB100–only” allele. “Apo-B100–only” LDLR^−/−^ mice have higher LDL cholesterol levels than LDLR^−/−^ mice on a chow diet and develop more severe atherosclerosis than that of apoE^−/−^ mice (Veniant et al., 1998, 2000). The hypercholesterolemia, in the Reversa mouse, can be eliminated by inducing the expression of the Mx1-Cre transgene, which inactivates the gene for microsomal triglyceride transfer protein (Mttp) that is required for the secretion of apoB-containing lipoproteins (Gordon et al., 1995; Lieu et al., 2003; Feig et al., 2011). We reasoned that the Reversa mouse could be used to study the regression of atherosclerosis if the hyperlipidemia were reversed later in life after plaques had developed. Using this model, we found that reversal of hyperlipidemia led to lower levels of CD68+ cells in plaques. Furthermore, the regression process was characterized by reduced plaque lipid and increased content of collagen. There were also changes in plaque CD68+ cells, including (1) evidence for migratory behavior *in vivo*; (2) decreased expression of genes encoding inflammatory and prothrombotic factors; and (3) enrichment in markers of the M2 macrophage state (Feig et al., 2011). These data suggest that there are certain features that are common to the regression process.

Cholesterol homeostasis has also recently been investigated with microRNAs (miRNA), which are small endogenous non–protein-coding RNAs that are posttranscriptional regulators of genes involved in physiological processes. MiR-33, an intronic miRNA located within the gene encoding sterol-regulatory element binding protein-2, inhibits hepatic expression of both ABCA1 and ABCG1, reducing HDL-C concentrations, as well as ABCA1 expression in macrophages, thus resulting in decreased cholesterol efflux (Rayner et al., 2011). Interestingly, enrichment of M2 markers in plaque CD68+ cells was observed in LDLR^−/−^ mice treated with an antagamir of miR-33 (Rayner et al., 2011). The treated mice also exhibited plaque regression (fewer macrophages and intimal area). The therapeutic potential of miR-33 antagmirs to cause similar benefits in people was suggested by plasma levels of HDL being raised in treated non-human primates (Rayner et al., 2011). Thus, antagonism of miR-33 may represent a novel approach to enhancing macrophage cholesterol efflux, raising HDL-C levels, and promoting regression.

## Conclusion

**Figure 1:**
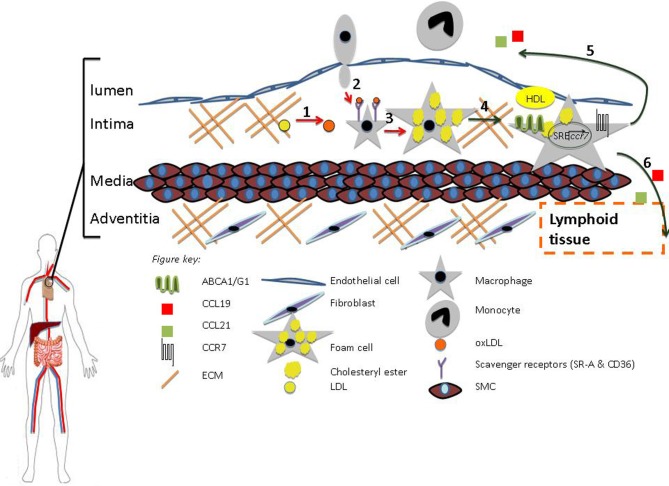
**HDL promotes regression.** (1) Oxidation (2) Diapedesis (3) Foam Cell formation (4) RCT (5–6) Macrophage Egress from Lesion to Lumen and Adventitia, respectively. HDL can inhibit processes 1–3 (red arrows) and promote 4–6 (green arrows). Macrophage egress can occur through the upregulation of CCR7 via activation of the sterol regulatory element binding protein (SREBP) pathway.

**Figure 2:**
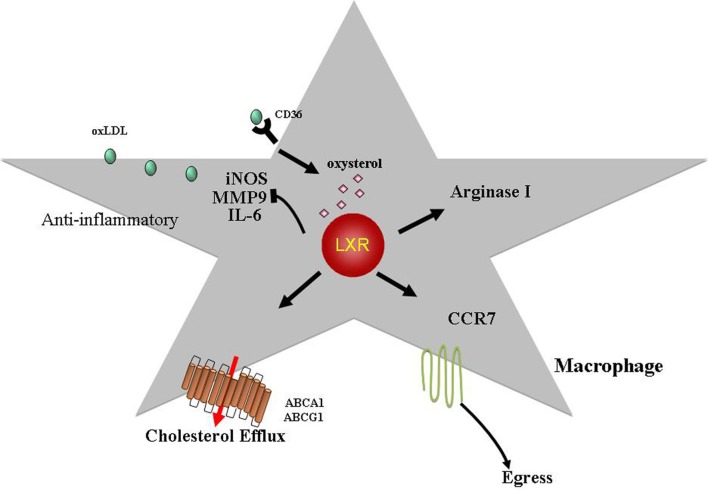
**LXRs exhibit anti-atherogenic properties.** LXR ligands, oxysterols, activate the LXR transcription factors (LXRα, LXRβ) leading to increased expression of targets such as ABCA1 and ABCG1. This is a key step in promoting cholesterol efflux from macrophages. LXRs have also been shown to inhibit expression of inflammatory mediators such as iNOS, MMP9, and IL6. Recently, it was demonstrated that LXRα activation can induce arginase I (M2 macrophage marker) expression suggesting that LXRs can skew macrophages toward a M2 phenotype (alternative activated, anti-inflammatory; see text for details). In addition, we have shown that LXRs are required for atherosclerosis regression and can induce CCR7 expression in macrophages with the resultant egress out of the lesion. Hence, in addition to promoting reverse cholesterol transport and having anti-inflammatory properties, LXRs can regulate the migratory potential of macrophages in the plaque making these transcription factors therapeutic targets against atherosclerosis.

It is quite clear that both macrophages and DCs play important roles in atherosclerosis. While a tremendous amount of progress has been made in elucidating how this is so, there are still many unanswered questions. The current evidence, however, suggests that macrophages and DCs have the ability to change their inflammatory, metabolic, and migratory properties. The data demonstrate that the regression of atherosclerosis is possible with factors such as HDL, LXR, and CCR7 playing key roles (Figures 1, 2). Ultimately, the insights gained from further experimentation will lead to new therapeutic targets against cardiovascular disease.

### Conflict of interest statement

The authors declare that the research was conducted in the absence of any commercial or financial relationships that could be construed as a potential conflict of interest.
